# A rare mushroom-shaped lesion growing in the transverse colon

**DOI:** 10.1055/a-2578-2558

**Published:** 2025-05-06

**Authors:** XiaoBo Liu, Jun Pan, TieYan Wang, ShengBao Li, Yuan-Jun Gao

**Affiliations:** 1 Department of Gastroenterology, Taihe Hospital, Hubei University of Medicine, Shiyan, China; 2 Department of Pathology, Taihe Hospital, Hubei University of Medicine, Shiyan, China

A 48-year-old woman visited our outpatient department after an outpatient colonoscopy revealed a mushroom-shaped protrusion of Yamada III type in the transverse colon, approximately 60 cm from the anus. It had a diameter of about 8 mm and no surface erosion. However, the center had a shallow depression ( Fig. 1 ). Her outpatient pathological report suggested that the tissue collected was heterotopic gastric mucosa (HGM). When we used an injection needle for submucosal injection, the surrounding area of the tumor was easily lifted, but lifting of the central depression was unsatisfactory ( Fig. 2 **a** ). After peeling off the mucosal and submucosal layers, we found that this lesion had a thick pedicle, and the root of this pedicle was located in the intrinsic muscle layer and grew toward the serosal layer ( Fig. 2 **b** , Video 1 ). The postoperative sample looked like a complete mushroom, with a size of about 2.0 × 2.5 cm. After completely removing the lesion, a droplet-shaped pit was left in its original position ( Fig. 2 **c** ).

**Fig. 1:**
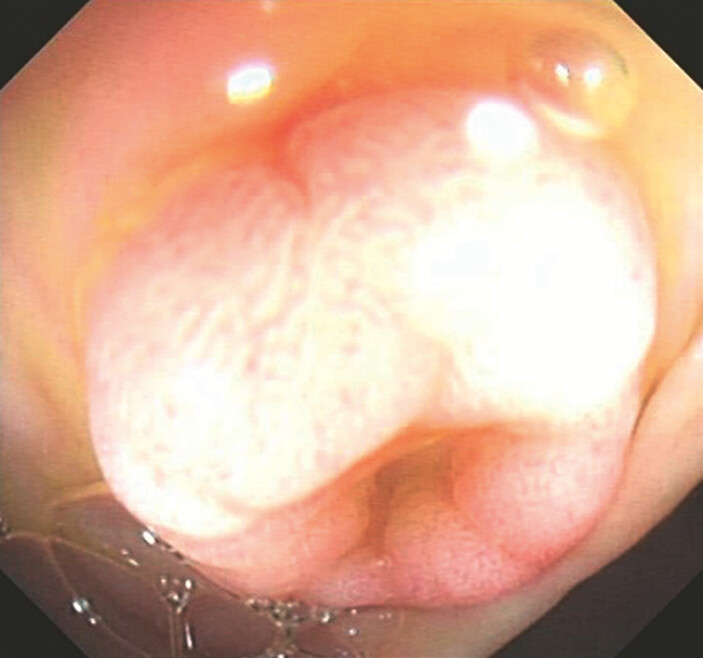
Endoscopic appearance of the colonic lesion: a mushroom-shaped protrusion.

**Fig. 2:**
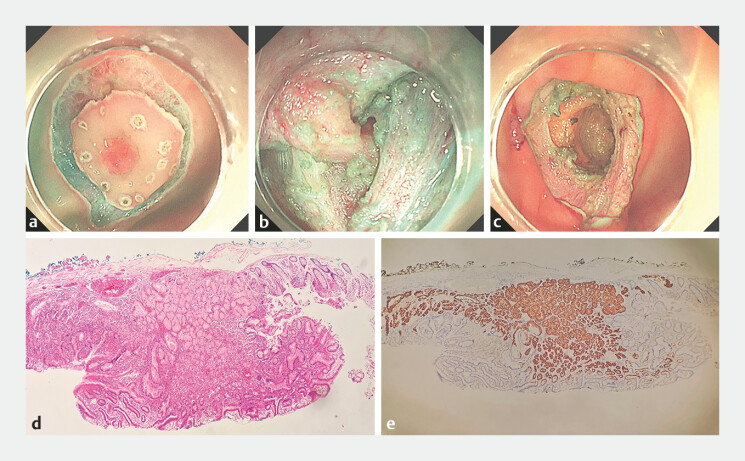
Endoscopic submucosal dissection (ESD) and postoperative pathological results. **a** The surrounding area of the tumor was easily lifted, but lifting of the central depression was unsatisfactory. **b** The root of the pedicle was located in the intrinsic muscle layer and grew toward the serosal layer. **c** A droplet-shaped pit was left in its original position. **d** Heterotopic gastric mucosa in the ESD postoperative specimen (hematoxylin and eosin, original magnification ×10). **e** Immunohistochemistry confirmed positivity for MUC6.

Endoscopic submucosal dissection for removal of gastric mucosal ectopia originating from the intrinsic muscle layer of the transverse colon.Video 1

Under hematoxylin and eosin staining microscopy, ectopic gastric mucosal tissue was observed on the left and middle parts, presenting as a polyp-like protrusion with a surface covered by gastric pit epithelium. The right side had a colonic mucosal epithelium rich in goblet cells, and the crypt structure was preserved ( Fig. 2 **d** ). Immunohistochemistry showed that MUC5AC was expressed in the gastric pit epithelium and MUC2 was expressed in the normal colonic mucosal epithelium. MUC6 was expressed in the gastric pyloric glandular tissue ( Fig. 2 **e** ), confirming HGM in the transverse colon.

HGM can appear throughout the digestive tract and is relatively rare in the colon. Val-Bernal et al. 1 reported a case of a polyp located 50 cm from the anal margin, and Ito et al. 2 reported a case of colonic ectopic gland, a submucosal tumor located in the transverse colon with a raised center surrounded by a fissure. To the best of our knowledge, our study is the first case of HGM with a mushroom-like appearance appearing in the transverse colon, and the mass affected almost the entire intestinal wall.

Endoscopy_UCTN_Code_TTT_1AQ_2AD_3AD

## Notes

Endoscopy E-Videos https://eref.thieme.de/e-videos
*E-Videos* is an open access online section of the journal *Endoscopy* , reporting on interesting cases and new techniques in gastroenterological endoscopy. All papers include a high-quality video and are published with a Creative Commons CC-BY license. Endoscopy E-Videos qualify for HINARI discounts and waivers and eligibility is automatically checked during the submission process. We grant 100% waivers to articles whose corresponding authors are based in Group A countries and 50% waivers to those who are based in Group B countries as classified by Research4Life (see: https://www.research4life.org/access/eligibility/ ). This section has its own submission website at https://mc.manuscriptcentral.com/e-videos .

## References

[1] Val-Bernal J Cagigal M Mayorga M Colonic tubular adenoma with incidental oxyntic gastric heterotopia Rom J Morphol Embryol 2021 62 313 318 34609438 10.47162/RJME.62.1.35 PMC8597387

[2] Ito S Hotta K Imai K Endoscopic submucosal dissection for a heterotopic gland in the transverse colon Endoscopy 2017 49 E288 E289 10.1055/s-0043-117942 28905334

